# E2-EPF UCP regulates stability and functions of missense mutant pVHL via ubiquitin mediated proteolysis

**DOI:** 10.1186/s12885-015-1786-8

**Published:** 2015-10-26

**Authors:** Kyeong-Su Park, Ju Hee Kim, Hee Won Shin, Kyung-Sook Chung, Dong-Soo Im, Jung Hwa Lim, Cho-Rok Jung

**Affiliations:** Gene Therapy Research Unit, KRIBB, Daejeon, Republic of Korea; Genome research center, KRIBB, Daejeon, Republic of Korea; University of Science and Technology, Daejeon, Republic of Korea; EQUISnZAROO R&D center, Gyeonggi-do, Republic of Korea

**Keywords:** VHL disease, pVHL missense mutation, E2-EPF UCP, Ubiquitination, Protein instability

## Abstract

**Background:**

Missense mutation of *VHL* gene is frequently detected in type 2 VHL diseases and linked to a wide range of pVHL functions and stability. Certain mutant pVHLs retain ability to regulate HIFs but lose their function by instability. In this case, regulating of degradation of mutant pVHLs, can be postulated as therapeutic method.

**Method:**

The stability and cellular function of missense mutant pVHLs were determine in HEK293T transient expressing cell and 786-O stable cell line. Ubiquitination assay of mutant VHL proteins was performed in vitro system. Anticacner effect of adenovirus mediated shUCP expressing was evaluated using ex vivo mouse xenograft assay.

**Results:**

Three VHL missense mutants (V155A, L158Q, and Q164R) are directly ubiquitinated by E2-EPF UCP (UCP) in vitro. Mutant pVHLs are more unstable than wild type in cell. Missense mutant pVHLs interact with UCP directly in both in vitro and cellular systems. Lacking all of lysine residues of pVHL result in resistance to ubiquitination thereby increase its stability. Missense mutant pVHLs maintained the function of E3 ligase to ubiquitinate HIF-1α in vitro. In cells expressing mutant pVHLs, Glut-1 and VEGF were relatively upregulated compared to their levels in cells expressing wild-type. Depletion of UCP restored missense mutant pVHLs levels and inhibited cell growth. Adenovirus-mediated shUCP RNA delivery inhibited tumor growth in ex vivo mouse xenograft model.

**Conclusion:**

These data suggest that targeting of UCP can be one of therapeutic method in type 2 VHL disease caused by unstable but functional missense mutant pVHL.

**Electronic supplementary material:**

The online version of this article (doi:10.1186/s12885-015-1786-8) contains supplementary material, which is available to authorized users.

## Background

The von Hippel-Lindau (VHL) disease is caused by mutation of *VHL* tumor suppressor gene and classified into two types depend on genotype-phenotype correlation. The mutation of Type 1 VHL disease is truncation or exon deletion and type 2 VHL disease have missense mutation commonly. Type 2 VHL disease shows a high risk of pheochromocytoma (PCC) and germ line missense mutations is subdivided into high risk (2B), low risk (2A), or absence (2C) of Renal cell carcinoma (RCC) and heamangioblastoma is correlated with function of pVHL to impair HIF-1α activity [1, 2]. Regarding to HIFs regulation, type 1 and type 2B VHL disease have high defect and type 2A relative low defect. In certain types 2VHL disease, mutations of *VHL* gene retain their functionality to regulating HIFs but they exhibit instability of mutant VHL protein [3–5]. However the mechanisms control the instability of missense mutant pVHLs are still under discovered.

Proteasome dependent proteolysis is efficient and powerful system for regulating half-life of cellular proteins. Ubiquitination is start signal for proteasomal degradation which is consisted by E1, E2 and E3 enzyme. pVHL is the substrate recognition component of an E3 ubiquitin ligase complex that also contains elongin B, elongin C, Cul2, and Rbx1 [6–9]. pVHL has two functional domains that directly bind to elongin C and pVHL substrates, respectively and it targets the HIFs for ubiquitin-mediated degradation [5, 10–13]. Prolyl-hydroxylated HIFs are recognized by pVHL, which results in it being polyubiquitinated and, thereby, targeted for proteasomal degradation [14, 15]. The different domains of pVHL are also important for its stability because mutant pVHL which are defective in elongin C binding, are unstable and are rapidly degraded [16]. pVHL also has role in maintaining extracellular matrix (ECM) thus pVHL-knock out cells like 786-O or RCC4 revealed loss of assembling fibronectin. The function of pVHL maintaining ECM is not depend on HIFs [17].

Human E2-EPF UCP (UCP) was the first E2 family member to be cloned from epidermal tissue [18]. Expression of UCP is five times higher in common human cancers than in normal tissues [19, 20] Roos et al. has been reported that UCP implicated in papillary RCC which is second most common subtype of kidney cancer [21]. Recombinant UCP is a bifunctional enzyme that is capable of catalyzing E3-independent and E3-dependent ligation of ubiquitin and UCP targets pVHL for ubiquitin-mediated degradation [22, 23]. Since UCP impair to tumorigenesis, we examined whether UCP can degrade V155A, L158Q and Q164R missense mutant pVHLs which are linked to RCC. In this study, new biochemical mechanism of instability of missense mutant pVHL is provided and UCP can be served as a therapeutic target for RCC which is related missense mutation of *VHL* gene.

## Methods

### Antibodies and reagents

Anti-Flag, anti-GST and anti-b-actin antibodies were purchased from SIGMA-Aldrich. Anti-HA antibody was purchased from AbFrontier, and anti-His antibody was purchased from Millipore. Human anti-HIF-1α was purchased from BD Pharmingen, and human anti-HIF-2α was purchased from Santa Cruz Biotechnology. The anti-UCP antibody was generated by protocol, as reported previously [23]. The proteasome inhibitor MG132 was purchased from Boston Biochem, and cycloheximide was purchased from SIGMA-Aldrich. Luminol assay kit was purchased from Promega.

### Plasmids

Human UCP, elongin C, HIF1a, and UbcH5C cDNA molecules were supplied by the 21C Frontier Human Gene-Bank, South Korea. Full-length UCP was cloned into pET28a (novagen) and pCMV-tag1 (Stratagene). Wild-type pVHL and point mutants were cloned by PCR amplification from pFlag-VHL (a gift from S. Cho, Chung-Ang University, Seoul, South Korea) into pCDNA3.1+ (Invitrogen), pEBG, pGEX-4 T1 and pET-28a. The shUCP (5′-AATGGCGATCTGCGTCAAC-3′) sequence was inserted into the pSUPER vector according to the manufacturer’s instructions (Invitrogen). The sequences of all plasmids were verified by direct sequencing before use. pTK-Hyg (Clontech) was used for producing HeLa -shUCP expressing constitutive cell line. Five repeat copies HRE derived from VEGF promoter cloned to pGL3 (Promega).

### Cell culture and counting

786-O cells, HEK293T cells and HeLa cells were maintained in Dulbecco’s modified Eagle’s medium (DMEM) with 10 % fetal bovine serum (FBS, GIBCO) in a humidified incubator with 5 % CO2 at 37 °C. The 786–O cell lines stably expressing exogenous pVHL were transfected with the indicated plasmids or empty vector (pCDNA3.1), and were cultured with 1 mg/mL geniticin (G418, GIBCO) for 1 month for single colony selection. For the cell proliferation assay, the cells were plated at 5 × 10^3^ cells/well in conditioned media on 24-well plates. At 24 h after seeding, the cells were trypsinized and counted by a hemocytometer. The viability of cells were observed by crystal violet staining (0.1 % w/v). Luminol assay for HRE-luc was performed as manufacturer’s indication.

### Protein stability analysis

The 786-O cell lines stably expressing exogenous HA-tagged wild-type or mutant pVHLs were treated with 50 μg/ml cycloheximide for 0, 2, 4 and 6 h. At the indicated time points, the cells were harvested, and proteins were detected by western blot analysis with a VHL antibody (BD Pharmiongen). The signal intensity was determined using densitometer software.

### Immunoblot analysis and pull down assay

Cells were lysed on ice using RIPA buffer (50 mM Tris-HCl, pH 7.5, 150 mM NaCl, 0.5 mM EDTA, 1 % NP40, 0.1 % SDS, 1 mM PMSF, 1X protease inhibitor) and were separated by 12 % SDS-PAGE. The proteins were transferred from the gel onto a PVDF membrane (polyvinylidene fluoride, Millipore), and the membrane was incubated with specific primary antibodies in PBS/0.1 % Tween20 (PBST) for 2 h at RT or overnight at 4 °C. Subsequently, the membrane was incubated with secondary antibody in PBST containing 0.5 % skim milk for 1 h at RT, and the proteins were visualized using a chemiluminescence kit (Intron). The cell lysate was prepared in NET gel buffer (50 mM Tris-HCl, pH 7.5, 150 mM NaCl, 0.1 % NP-40, 1 mM EDTA, pH 8.0) supplemented complete proteinase inhibitor cocktail (Roche), and GST-tagged and His-tagged proteins were pulled-down with the glutathione sepharose beads (GE healthcare) and Ni-NTA agarose (Qiagen). Proteins were separated by SDS-PAGE and detected by immunoblot with antibody as indicated.

### Purification of recombinant fusion proteins

GST fusion proteins were expressed and purified as described by the manufacturer (Amersham). pGEX-4 T1 vector-based GST fusion proteins were induced with 1 mM IPTG for 2 h at 37 °C. Cells were washed with PBS, resuspended in lysis buffer (PBS, protease inhibiter cocktail, 1 mM PMSF), and then sonicated on ice. Soluble protein extracts were added to glutathione sepharose 4B resin (Amersham) and incubated for 2 h at 4 °C. The columns were washed five times with PBS. Bead-bound proteins were eluted with elution buffer (50 mM Tris, pH 8.8, 1 mM EDTA, 20 mM glutathione reduced (GSH), 1 mM PMSF). His-fusion proteins were expressed and purified as described by the manufacturer (QIAGEN). pET28a vector-base His fusion proteins were induced with 1 mM IPTG for 2 h at 37 °C. The cells were resuspended in lysis buffer (0.5 M NaCl, 5 mM imidazole, 20 mM Tris, pH 7.9) and then sonicated on ice. The cell extracts were added to Ni-NTA resin (QIAGEN) and were incubated for 2 h at 4 °C. The columns were washed five times with wash buffer (0.5 M NaCl, 60 mM imidazole, 20 mM Tris, pH 7.9). Bead-bound proteins were eluted with elution buffer (0.25 M NaCl, 0.5 M imidazole, 10 mM Tris, pH 7.9). After dialysis, the purified proteins were stored at -70 °C.

### Ubiquitination assay

In vivo ubiquitination assay was performed by protocol, as previously described [23]. For the self-ubiquitination of UCP, the reaction mixture (50 μl) contained 0.3 μg of GST-UCP, 0.5 μg of His-E1 and 25 μg/ml Flag-ubiquitin in reaction buffer (25 mM Tris-Cl, pH 7.5, 1 mM ATP, 5 mm creatine phosphate, 0.5 μg/ml creatine phosphate kinase, 1 mM DTT, 5 mM MgCl_2_, 0.5 μg/ml ubiquitin aldehyde) was used. The mixture was incubated for 1 h at 37 °C, and then a western blot analysis was performed using the indicated antibodies. For the ubiquitination of pVHL by UCP, the reaction mixture (50 μl) containing 0.3 μg of GST-UCP, 0.5 μg of His-E1, 3 μg of His-VHL, and 25 μg/ml Flag-ubiquitin in reaction buffer was used. After incubation at 37 °C for 1 h, GST-VHL was pulled down with glutathione sepharose 4B resin and was analyzed by SDS–PAGE. For the ubiquitination of HIF-ODD by the VHL-elongin B-elongin C (VCB) complex, the 786-O cells were washed and collected in PBS. The cells were disrupted, using a sonicator, in lysis buffer (50 mM Tris-HCl, pH7.5, 150 mM NaCl, 0.5 mM EDTA, 0.1 % NP40, 1 mM PMSF, 1X protease inhibitor). The cell lysates were centrifuged at 13000 rpm for 1 h at 4 °C. The total reaction volume was 50 μl and contained 50 μg of 786-O cell extracts, 3 μg of GST-ODD, 0.3 μg of His-VHL, 0.3 μg of UBCH5C, and 0.5 μg of His-E1 in reaction buffer. The mixtures were incubated for 2 h at 30 °C. After incubation, the reaction mixtures were pulled down with glutathione sepharose 4B resin and analyzed by SDS–PAGE.

### RT-PCR and real time PCR

Total RNA was extracted from cells using an easy-spin RNA extraction kit (Intron). Complementary DNA (cDNA) was synthesized using 3–5 μg of total RNA, reverse transcriptase (TakaRa, Japan) and oligo (dT) primer. cDNA was amplified by polymerase chain reaction using primers specific for each gene (Additional file 5: Table S1). For the LightCycler (Roche Diagnostics) reaction, LightCycler mastermix and cDNA as the PCR template were filled in PCR tube. The mixtures were centrifuged and placed into the LightCycler rotor. The following LightCycler experimental run protocol was used: denaturation (95 °C for 10 min), amplification and quantification repeated 35 times (95 °C for 15 s, 60 °C for 10 s, and 72 °C for 60 s) with a single fluorescence measurement.

### Animals and ex vivo xenograft assay

Seven-week-old female BALB/c nude mice were purchased from SLC japan and maintained in a accordance with guidelines and approval of Institutional Review Committees for Animal Care and Use, Korea Research Institute of Bioscience and Biotechnology (KRIBB-AEC-14024). 786-O and 786-VHL (WT and V155A) cells were transduced with adenoviral vectors (Ad.shUCP and Ad.shCont) at 200 MOI for 24 h. And then cells (10^7^) are transplanted by subcutaneous injection into nude mice (Japan SLC, Inc.). Tumor size was measured for 44 days by following procedure, as reported previously [23, 24].

### Statistics

Statistical analysis was carried out using the unipolar, paired Student *t* test and the two-sided chi-square test. Data were considered statistically significant when the *P* value was less than 0.05.

## Results

### Protein instability of missense mutant pVHL is caused by proteasome dependent degradation

To examine missense mutations of VHL gene associated with type 2 VHL disease, we selected total 7 VHL missense mutants which were characterized as tumorigenic cluster. Missense mutation like V155A, L158Q, Q164R, R167Q and L188V were located on α-domain which interacted to Elongin C. Mutants such as N78S and Y112H were involved in role for recognition of HIF-1 α protein (Additional file 1: Figure S1A). UCP was found to ubiquitinate all of them in vitro ubiquitination assay using recombinant missense mutant pVHL (Additional file 1: Figure S1B).

**Fig. 1:**
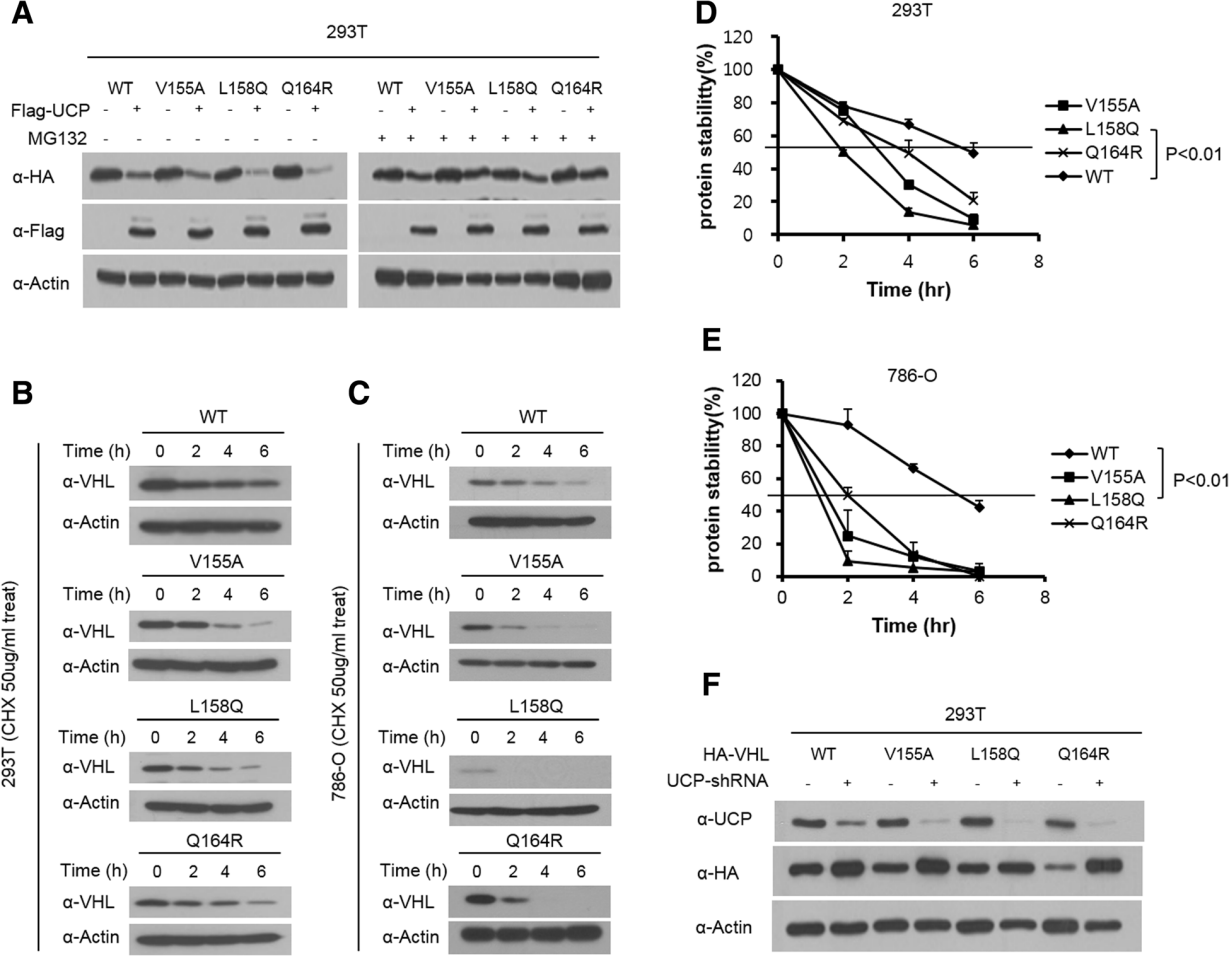
UCP degraded missense mutant pVHLs (V155A, L158Q and Q164R) via proteasome pathway and decrease half-life in cell. **a** HEK293T cells were transfected with HA-tagged mutant *VHL* (V155A, L158Q, Q164R) and/or Flag-UCP and cells were incubated for 16 h either in the presence or absence of 10 uM MG132 at 36 h post- transfection. **b** Mutants *VHL* were transfected into 293 T cell. At 36 h post- transfection. Cells were treated with cyclohexamide (CHX) for 0, 2, 4, 6 h then and immunoblotted as indicated. **c** 786-O cell lines constitutively expressing mutant pVHL were treated with cyclohexamide (CHX) for 0, 2, 4, 6 h then and immunoblotted as indicated. Calculation of protein degradation kinetic of both HEK293T (**d**) and 786-O (**e**) cell lines was revealed that mutant pVHLs have shortened half-life (*n* = 3, *p* < 0.01). **f** HEK293T cells were transfected with HA-tagged mutant *VHL* and/or UCP-shRNA and then at 48 h post- transfection, cell were harvested and immunoblotted as indicated

We examined whether UCP regulated the stability of the selected three missense mutant pVHLs. HEK293T cells were transfected with the Flag-UCP plasmid and each mutant VHL plasmid respectively, either in the presence or absence of MG132, a proteasome inhibitor (Fig. 1a). Missense mutant pVHLs were degraded by UCP, but this was inhibited by MG132. Then, we confirmed the changing half-life of the mutant pVHLs using cycloheximide (CHX)-mediated pulse chase assay in both HEK293T (Fig. 1b) and 786-O cells (Fig. 1c). The three mutant pVHLs had shorter half-life than the wild type pVHL in both cell lines (Fig. 1d and e). A relatively higher transfection efficiency was observed for HEK293T cells than for 786-O cells, and this led to an increased half-life of mutant pVHLs in HEK293T cells as compared to 786-O cells (Fig. 1b and c). Furthermore, the L158Q mutant was so unstable in 786-O cells that it was nearly undetectable (Fig. 1c). We examined that the protein levels of the mutant pVHLs were dependent on the UCP levels in the cells. We co-transfected the VHL missense mutants with the shUCP plasmid into HEK293T cells and confirmed that UCP depletion increased the levels of the missense mutant pVHL (Fig. 1f). These result led us to conclude that UCP regulates the stability of missense mutant pVHL.

### Missense mutations of VHL gene associated with RCC were targeted by UCP directly in vitro and in vivo

**Fig. 2:**
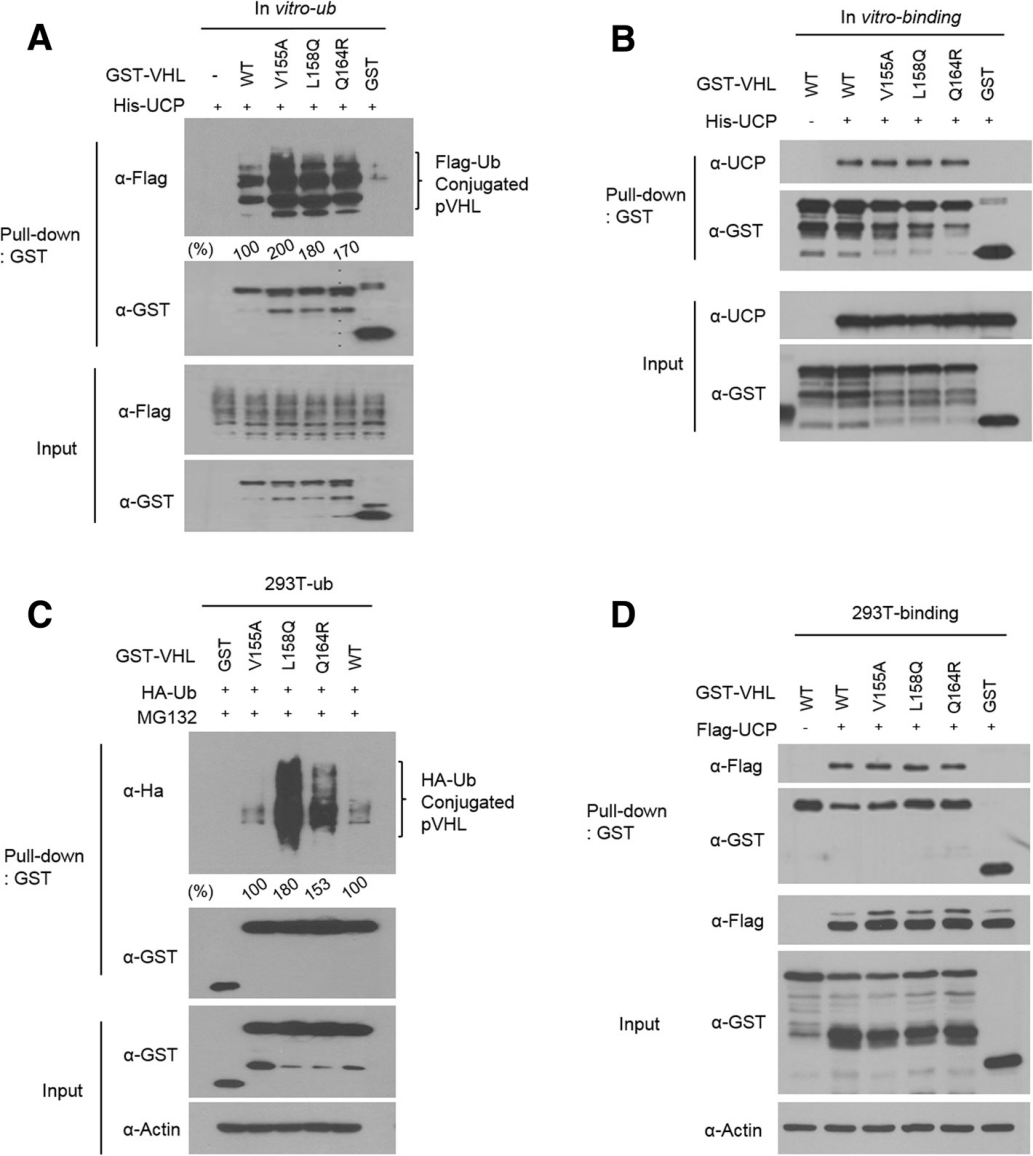
UCP interacted missense mutant pVHLs directly and ubiquitinated in vitro and in cell. **a** GST tagged recombinant proteins of mutant pVHLs were ubiquitinated by UCP in vitro. GST-pVHL (V155A, L158Q, Q164R) and/or His-UCP protein were incubated at 37 °C in the presence of E1 and Flag-ubiquitin. GST-pVHL polyubiquitination was detected by western blot with Flag antibody. **b** Each mutant GST-VHL protein and His-UCP protein were mixed and then detected the interaction using GST-pull down assay. Bound UCP protein was detected by immunoblot. **c** Plasmid expressing GST tagged mutant *VHL* were transfected into HEK293T in the presence of MG132 for 16 h and then ubiquitinated forms were isolated using GST resin and then detected by immunoblot using antibody to detect HA-Ub conjugated pVHL. **d** Plasmid expressing GST tagged mutant *VHL* and /or Flag-UCP were transfected into HEK293T in the presence of MG132 for 16 h and then mixed and then detected the interaction using GST-pull down assay. Bound UCP protein was detected by immunoblot

Recombinant proteins of three missense mutants (V155A, L158Q and Q164R) were purified and provided to UCP as substrates for polyubiquitination assay. UCP bound and ubiquitinated all mutant pVHLs (Fig. 2a and b). In the ubiquitination assay, UCP generated ubiquitin chains on the mutant pVHLs more easily than on wild-type pVHL. UCP also recognized mutant pVHLs and ubiquitinated them in a cellular system (Fig. 2c and d). Interestingly, the L158Q mutant was the most highly ubiquitinated mutant in the cellular system. GST-tagged pVHL mutants were co-transfected into HEK293T cells with a HA-tagged ubiquitin plasmid, and then, the cells were exposed to the proteasome inhibitor MG132 for 12 h. The ubiquitinated mutant pVHLs were pulled down by GST resin and detected by anti-HA antibodies. The results of both in vitro and in cellular assays suggested that missense mutations in VHL gene do not alter the protein folding structure that is necessary for its interaction with UCP.

### Altering pVHL lysine residues increases the life span in a cellular system

**Fig. 3:**
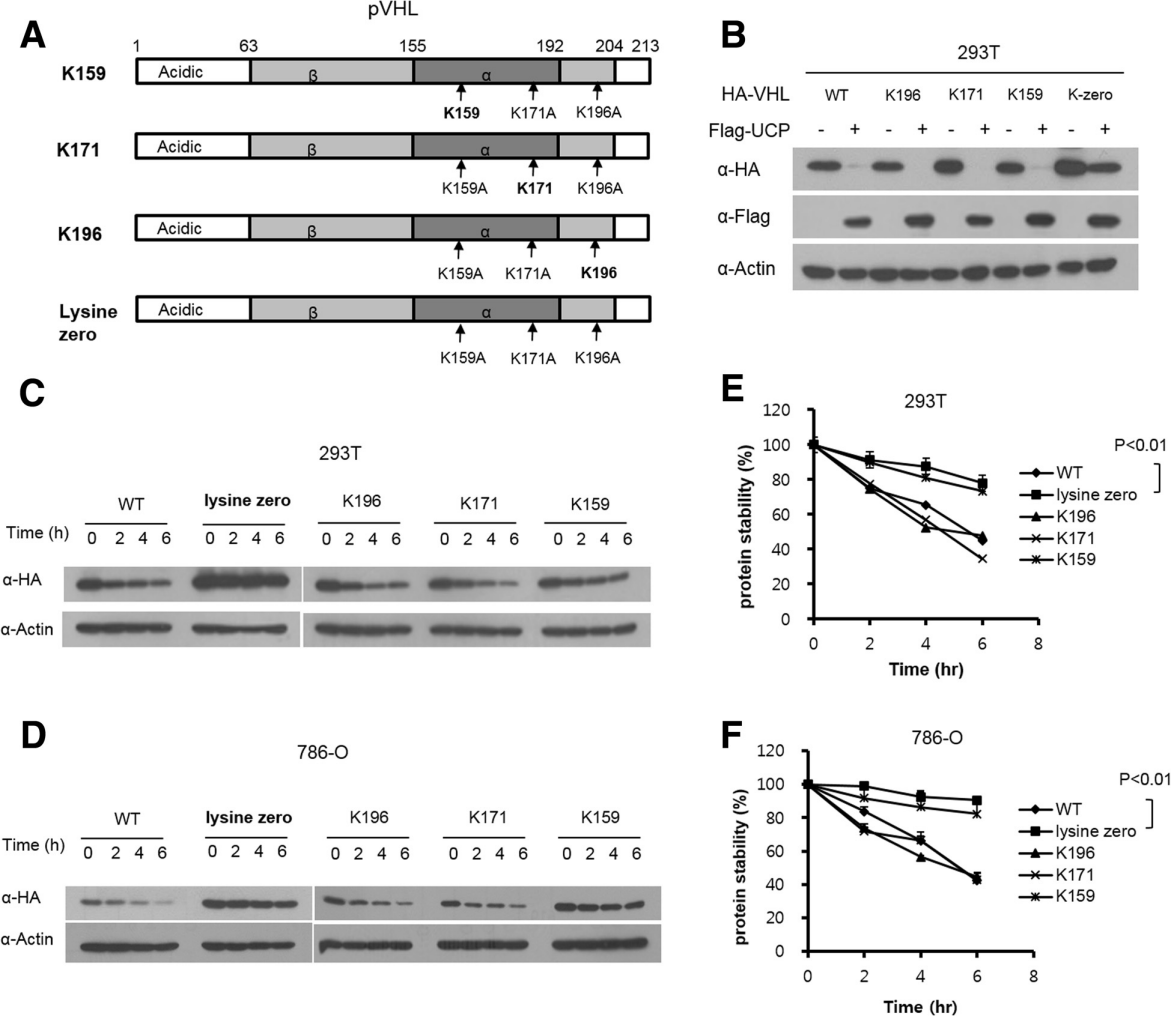
Lysine deficient mutant pVHL were resistant to degradation therefore increased half-life. **a** Schematic diagrams for various lysine deficient VHL mutants used in this study. **b** HA tagged lysine deficient mutant *VHL* were transfected into HEK293T and/or Flag-UCP. At 48 h post-transfection, cells were harvested and analyzed by western blotting. **c** Each lysine mutated pVHL expressing plasmids were transfected in HEK293T. At 36 h post- transfection. Cells were treated with cyclohexamide (CHX) for 0, 2, 4, 6 h then and immunoblotted as indicated. **d** 786-O cell lines constitutively expressing lysine mutant (K159, K171, K196, Lysine zero) stable cell lines were treated with cyclohexamide (CHX) for 0, 2, 4, 6 h then and immunoblotted as indicated. Calculation of protein degradation kinetics of HEK293T (E) and 786-O (**f**) cell lines showed that half-life of lysine deficient pVHL were increased (*n* = 3, *p* < 0.01)

Ubiquitin chain elongation formed at the lysine residue of the substrate with certain linkage. The protein of *VHL* has three lysine residues (K159, K171 and K196), therefore we hypothesized that the polymerization of ubiquitin by UCP occurred at the lysine residues of pVHL (Fig. 3a). We examined the stability of pVHL mutants with alanine substitutions at 1 or more of pVHL’s lysine residues. A mutant with a double alanine substitution for lysine (K159, K171 and K196) and a lysine zero mutant (deletion of all lysine residues) were constructed into a mammalian expression vector to examine their degradation in a cellular system. HA-tagged lysine mutant VHL plasmids and a Flag-tagged UCP plasmid were co-transfected into HEK293T cells and then protein levels were analyzed by a western blot assay (Fig. 3b). Since UCP ubiquitinated both pVHL and single lysine mutant but not lysine zero mutant pVHL (Additional file 3: Figure S3), the lysine zero mutant was resistant to the degradation by UCP (Fig. 3b). The levels of lysine altering mutant pVHLs were determined using a CHX-mediated assay, and the lysine zero mutant was more stable than the single lysine mutant in both HEK293T and 786-O cells (Fig. 3c and d). pVHL lysine zero mutant showed the longest half-life and pVHL K159 mutant showed relatively longer half-life than the other mutants (Fig. 3e and f). These data suggested that UCP formed polyubiquitin chain on lysine residue of pVHL such as other E3 ubiquitin ligase, thereby altering lysine residue of pVHL can be a way to evade degradation.

### Missense mutant pVHLs form the VBC complex and retain E3 ubiquitin ligase activity

**Fig. 4:**
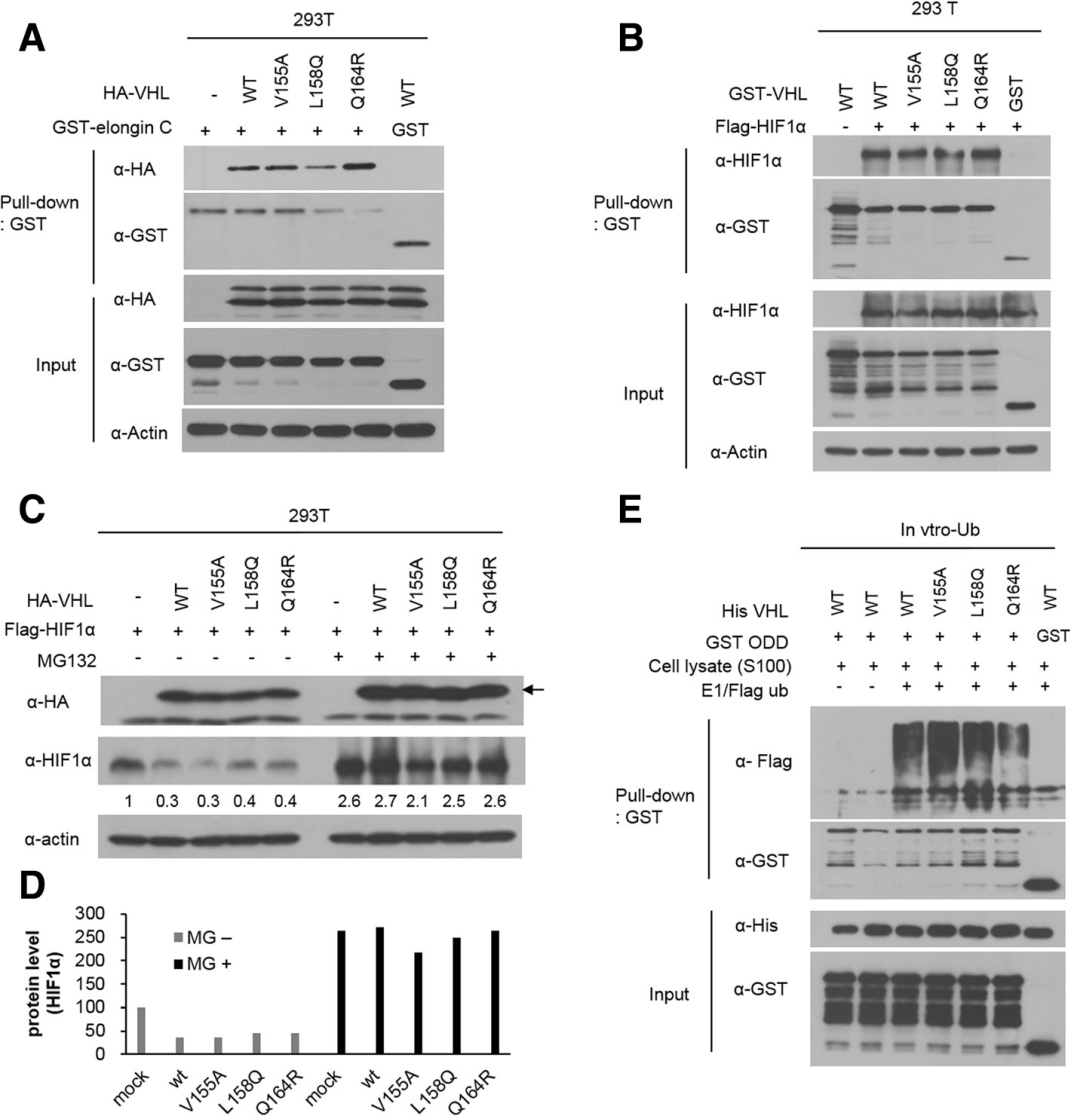
Missense mutant pVHL constitute E3 ubiquitin complex and retain functionality. **a** HEK293T cells were transfected with plasmid expressing HA tagged mutant *VHL* and/or GST tagged *Elongin C*. At 48 h post-transfection, cells were harvested and lysed in NET gel buffer and then detected the interaction using GST-pull down assay. Interacted mutant pVHLs were detected by immunoblot as indicated. **b** HEK293T cells were transfected with plasmid expressing GST tagged mutant *VHL* and/or Flag tagged HIF-1α. At 48 h post-transfection, cells were harvested and lysed in NET gel buffer and then detected the interaction using GST-pull down assay. **c** HEK293T cells were transfected with HA-tagged mutant *VHL* and/or Flag-HIF. Cells were incubated for 16 h either in the presence or absence of 10 uM MG132 at 36 h post- transfection. Protein levels are detected by immunoblotting as indicated. **d** Calculation of levels of HIF-1α at figure C and showed mutant pVHL degraded HIF-1α in cells. **e** Recombinant missense mutant pVHLs and GST-ODD protein were purified for in vitro ubiquitination assay. Cell lysates from 786-O cell line were provided as supplier of VBC components. His-tagged VHL proteins were incubated with E1, Flag-ubiquitin and GST-ODD protein with cell lystate at 37 °C for 1 h. GST-ODD protein was pulled-down with glutathione sepharose beads and ubiquitinated forms were detected by immunoblot with Flag antibody

To examine the ability of missense mutant pVHLs to form the E3 ligase complex and recognize HIFs, we performed a GST-pull down assay. GST-tagged elongin C was transfected with 3 missense mutant pVHLs (V155A, L158Q and Q164R) into HEK293T cells. Because the mutated amino acids are located near the pVHL site which is known as the elongin C binding site, we hypothesized that these mutants would not be capable of binding elongin C. However, all of missense mutant pVHLs bound to elongin C (Fig. 4a). The L158Q missense mutant pVHL exhibited the weakest interaction affinity as compared to the affinity of wild-type pVHL. Next, we transfected GST-tagged VHL missense mutant plasmids with or without the Flag-tagged HIF-1 α expression vector into HEK293T cells and performed a GST-pull down assay to detect HIF-1α. Three of missense mutant pVHLs recognized HIF-1 α with the same affinity (Fig. 4b). All of missense mutant pVHLs except V158Q used in this study, can build VBC complex in overexpressing system (Additional file 2: Figure S2).

Active pVHL ubiquitinated HIFs and the polyubiquitination of HIFs led to proteasome-dependent degradation of HIFs by cell-induced proteasome-mediated proteolysis. As expected, pVHL missense mutants induced the proteasome-dependent proteolysis of HIF-1α, and this proteolysis was inhibited by MG132 (Fig. 4c and d). The functionalities of pVHL missense mutants were tested by an in vitro ubiquitination assay with the ODD domain of HIF. pVHL missense mutants formed the VBC complex and directly ubiquitinated HIF-1α in vitro (Fig. 4e).

### The pVHL missense mutants modulate the cellular function of HIFs

**Fig. 5:**
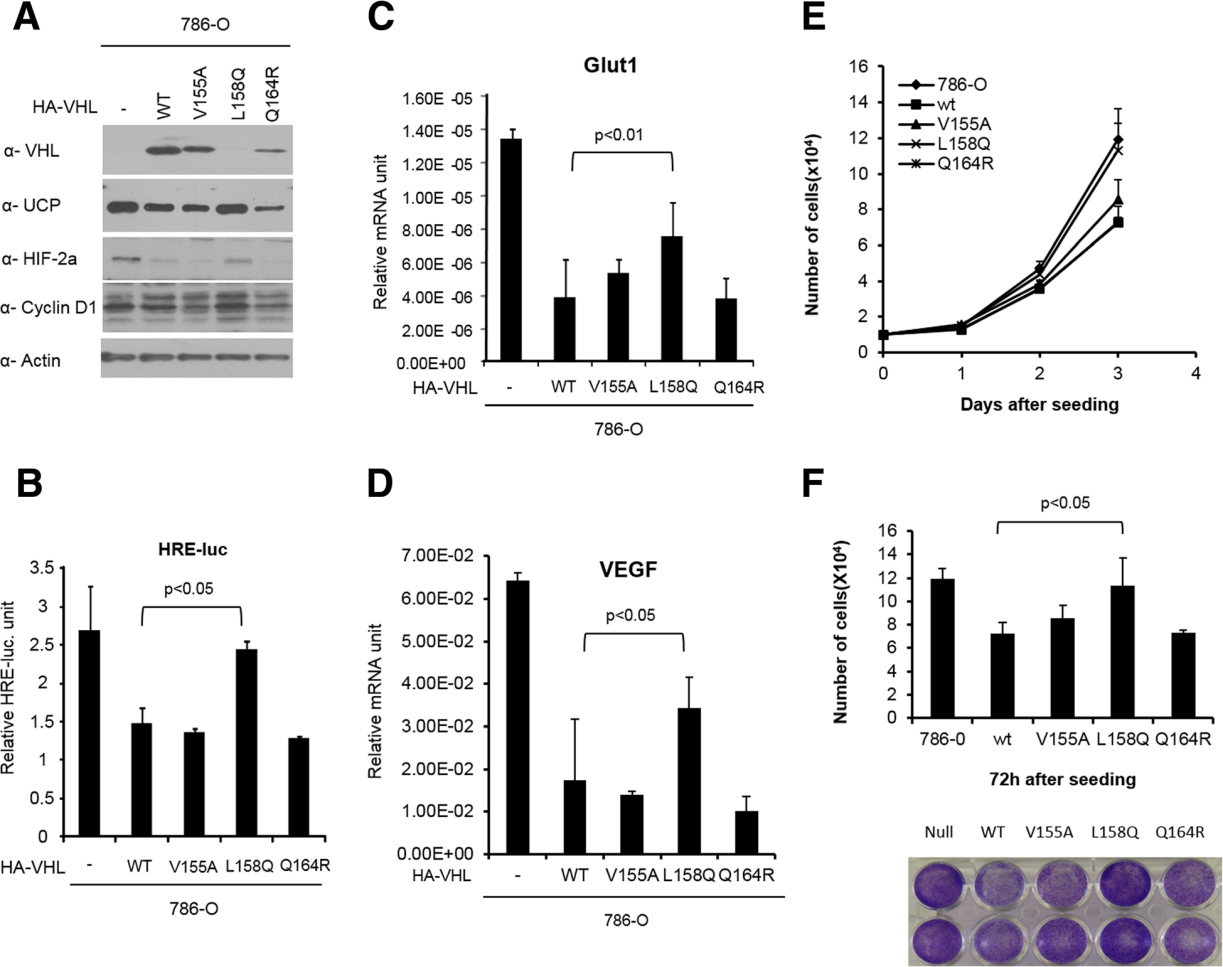
HIF-2α and its target genes was regulated by functional mutant pVHL. **a** HA tagged wild type and mutant *VHL* (V155A, L158Q, Q164R) were transfected into 786-O cells. At 48 h post-transfection, cells were lysed in RIPA buffer and then the proteins which are related in UCP-VHL-HIF pathway and cyclin D1 were detected by immunoblot as indicated. **b** HeLa expressing shVHL was transfected with HRE-Luc plasmid and/or missense mutant pVHL expressing plasmid. At 48 h post-transfection, cells were lysed in luminol assay buffer. Functionality of missense mutant pVHL was revealed by luciferase activity. mRNA was purified from missense mutant *VHL* expressing 786-O stable cell line at 48 h after seeding and it was used for quantitation of transcripts of Glut-1(**c**) (*n* = 3, *p* < 0.01) and VEGF (**d**) (*n* = 3, *p* < 0.05) using LightCycler. **e** Cell proliferative changes of each mutant pVHL expressing 786-O stable cell lines were observed by following 1, 2, 3 days using hemocytometric counting method. **f** The number and the conditions of cell at final days was observed by hemocytometric counting (*n* = 3, *p* < 0.05) and crystal violet staining

To examine whether missense mutant pVHLs modulate HIFs target genes, each missense mutant VHL gene was transfected into 786-O renal carcinoma cells, and then, HIF-2α and its targets were analyzed at the protein and mRNA levels. Missense mutant pVHL decreased the protein levels of HIF-2α and cyclin D1 (Fig. 5a) and the mRNA levels of Glut1 and VEGF were also decreased, as determined by real time PCR and conventional RT-PCR assays (Additional file 4: Figure S4). These events were dependent on the pVHL level; therefore, we concluded that missense mutant pVHLs regulated HIFs and its targets. The functionalities of missense mutant pVHLs were tested using a reporter assay with HRE-luciferase (Fig. 5b). We produced a HeLa cell line expressing shVHL, which depleted wild-type VHL using siRNA, and then, each mutant pVHL and pHRE-Luc was co-transfected into the cell line. Missense mutant pVHLs downregulated the promoter activity of HRE as well as the mRNA levels of Glut1 (Fig. 5c) and VEGF (Fig. 5d); however, L158Q showed little effect on the HRE promoter because it was expressed at a low level. These effects of the VHL mutation resulted in a decrease of the growth rate in the mutant-expressing 786-O cell lines. Wild-type pVHL showed the greatest reduction in growth rate, and L158Q did not affect cell growth (Fig. 5e–f). The same number of pVHL null 786-O cells and 786-O cells expressing missense mutant pVHL were seeded, and their cell numbers were counted every day for 3 days (Fig. 5e). The cell number at final day after seeding was counted and stained by crystal violet (Fig. 5f). We concluded that missense mutant pVHL (V155A, L158Q and Q164R) retain activity to regulate HIFs and the functionality was dependent on expression level of missense mutant pVHL.

### Depletion of UCP suppressed cell growth of the pVHL mutants in vitro and in vivo

**Fig. 6:**
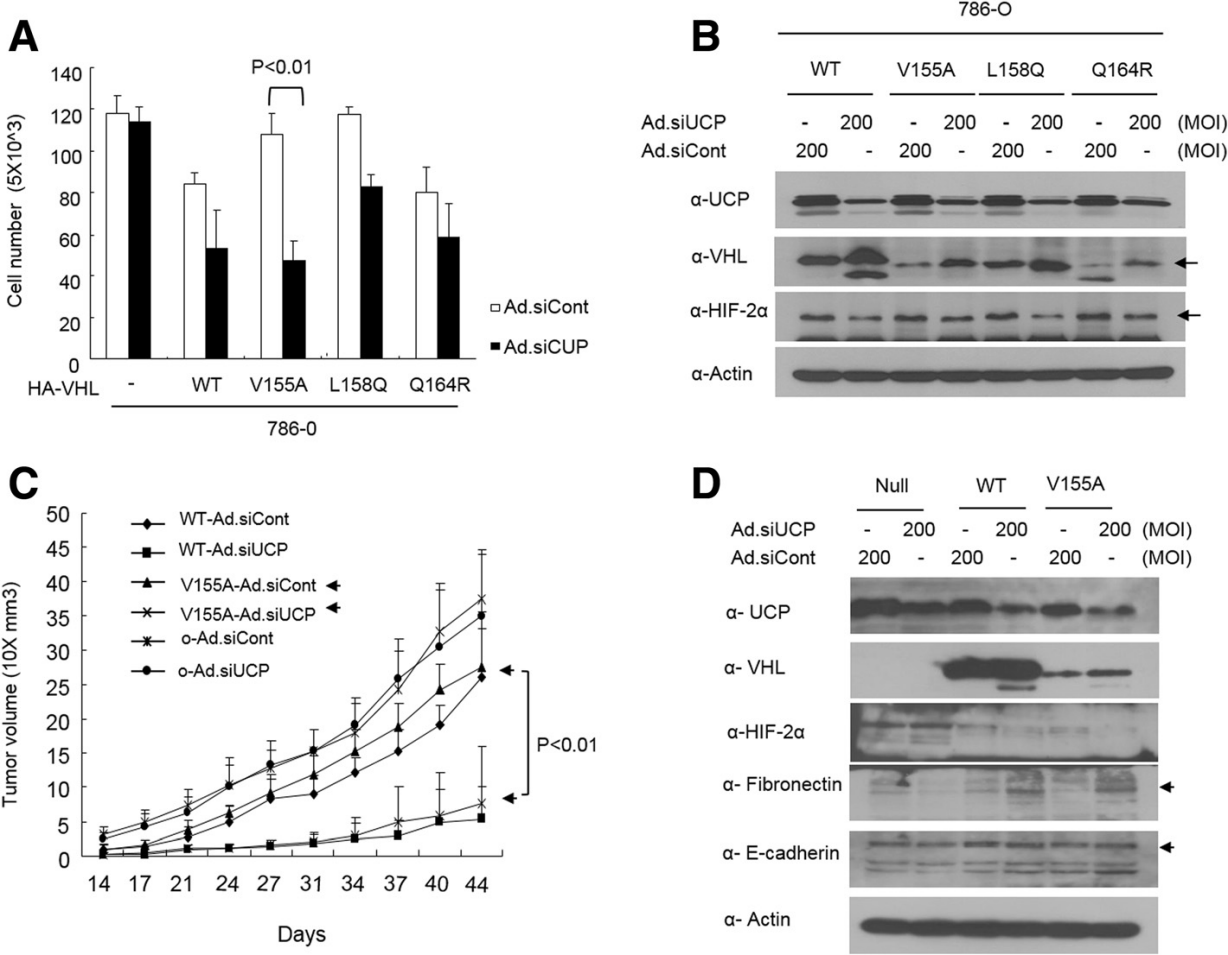
Gain of function for missense mutant pVHL using adenovirus mediated depletion of UCP. **a** V155A missense mutant pVHL expressing 786-O stable cell lines were transduced with adenovirus expressing siUCP or siControl at a MOI of 200 and incubated for 48 h. Cells were counted by hematocytometer (*n* = 3, *p* < 0.01) (**b**) At same time cells were harvested and lysed for analysis of UCP-VHL-HIF pathway. Proteins were monitored by immunoblot as indicated. **c** Cells as indicated were transduced adenovirus expressing UCP-siRNA and/or Cont-siRNA at 200 MOI and then injected into nude mouse subcutaneously (*n* = 4, *p* < 0.01). Tumor mass was monitored by 3-4 days during indicated times. **d** At the end of tumor size measurement, tumors were excised for immunoblot. Pooled tumor pieces of each group were lysed in tissue lysis buffer and immunoblot assay was performed as indicated

We tested whether UCP depletion could rescue VHL disease. First, we examined whether the growth rates of the cell lines expressing mutants pVHL were regulated by UCP. The 786-O cell lines expressing mutant pVHL were transduced by an adenovirus containing UCP-shRNA (Ad.shUCP) with 200 MOI. Cell growth was analyzed by the counting method, and protein levels were detected by the appropriate antibodies. Ad.shUCP suppressed cell growth in the cell lines expressing mutant pVHL, especially in the V155A missense mutant pVHL expressing 786-O cell line but not in the VHL null cell line (Fig. 6a). Ad.shUCP suppressed UCP expression and increased the level of pVHL, thereby decreasing the level of HIF-2α (Fig. 6b). An in vivo assay was conducted using a mouse xenograft model. The V155A missense mutant-expressing pVHL 786-O cell line and a VHL wild-type 786-O cell line were transduced with Ad.shUCP 200 MOI for 24 h and then transplanted on the skin of nude mice. Tumor growth was monitored for 44 days. On the last day of tumor measurement, the tumors were excised and then analyzed by western blotting. The inhibition of UCP result in decreasing the growth of tumors harboring the V155A missense mutant pVHL and the growth of tumors expressing wild-type VHL (Fig. 6c and d).

## Discussion

pVHL pocess E3 ubiquitin ligase activity to degrade HIFs which is related in tumor pomoting events but the mechanisms inducing instability of pVHL itself are not clarified clearly. Based on complex of VCB complex, folding and conformational chagnes of protein result in proteosomal degradation dependent on chaperones [16, 25]. A recent findings supported that missense mutant pVHL was easily degraded, and therefore had shortened half-life in cell [26, 27]. Missense mutation of *VHL* gene is most frequent in type 2 VHL disease. Depend on ability to control HIFs, it is classified into 2A, 2B and 2C. In case of type 2C, mutant pVHL retains function as E3 ubiquitin ligase to HIFs which induce angiogenic factors and stimulate glucose metabolism in cancer cells. These information suggest that inhibition or retardation of degrading pVHL is crucial for gain of function of missense mutant pVHL.

Based on the correlation between functional loss of pVHL and missense mutations in the VHL disease-associated tumors, VHL disease was classified into three clusters [24]. First cluster is formed by the surface residues are responsible for the interactions between elongin C and pVHL [10, 13]. The residues V155, L158, Q164 and R167 are the most frequently mutated residues in VHL syndrome [28]. The residue V155, L163 and V166 are associated in RCC [29]. The second cluster of mutations are located in HIFs protein binding site of pVHL binding [30]. Tyrosine 98 residue most popular mutated amino acid in this cluster that involved in tumorigenesis [31–33]. Last cluster of mutations are located on the β-domain and residues R79, S80, R82, L89, D121, Q132, L135, F136, and P138 are reported [34]. We characterized 7 VHL missense mutants as Y112H, R167Q, 188 V, V155A, L158Q, Q164R and N78S. Except V155A, 6 missense mutant pVHLs were discovered at nature and they are related with VHL disease [35–39].

UCP has been revealed as a factor that reconganize and targeted wild type pVHL for proteosomal degradation thereby stabilize HIFs. Depletion of UCP inhibit tumor growth and metastasis in vitro and in vivo and it is highly expressed in various cancer [23]. These findings lead us examine that UCP could recognize missense mutant pVHL and degrade it proteasome dependently like wild type pVHL and depletion of UCP level can rescue function of missense mutant pVHL. UCP was found to ubiquitinate all of missense mutant pVHL in vitro (Additional file 1: Figure S1) ubiquitination assay and these mutant pVHL interacted to HIF-1α (Additional file 2: Figure S2). These result suggested that tested mutant pVHL can regulate HIFs activity as far as it is stable. Taken together, UCP can be a critical factor for regulating HIFs via targeting missense mutant pVHL in RCC. In order to suggest meaning of UCP-VHL-HIFs axis, we characterized V155A, L158Q and Q164R missense mutant pVHLs which are most frequent in RCC. These three missense mutantations are located near the elongin C binding site of pVHL, which forms the pVHL-elongin complex to prevent the degradation of pVHL. UCP also recognized mutant pVHLs (V155A, L158Q and Q164R) and ubiquitinated them in vitro and in a cellular system. These missense mutations in *VHL* gene do not cause structural changes to the UCP binding site. Thereby UCP ubiquitinated missense mutant pVHLs, it caused degradation by proteasomes in cell. Ubiquitination by UCP can be a critical factor to determine stability of missense mutant pVHLs (Figs. 1 and 2). In addition to ubiquitination, some post translational modifications of protein by ubiquitn-like molecules like SUMOylation or NEDDylation has been reported as strategy for regulating protein dynamics in cells. What are major factors for regulating stability of missense mutant pVHL is still under question.

The polymerization of ubiquitin occurred at between glycine of the ubiquitin and the lysine residues of target protein. Ployubiquitin chain which is attached on lysine, recognized by 26S proteasome. UCP possess E3 ubiquitin ligase activity to wild type pVHL and VHL protein has three lysine residues (K159, K171 and K196). We hypothesized that UCP recognize three lysine of pVHL for ubiquitination. As expectation, pVHL lysine zero mutant had longest half-life and K159 mutant pVHL was relative long half-life than the others (Fig. 3). Indeed UCP mostly did not ubiquitinated lysine zero mutant pVHL in cell (Additional file 3: Figure S3). But there is significant difference of ubiquitination between single lysine mutant (Additional file 3: Figure S3). Therefore, K159 mutant pVHL is regulated by another mechanism in addition to ubiquitination. These results suggest that the inhibition of UCP mediated poly-ubiquitin chain elongation at the lysine residues of pVHL increased the stability of pVHL in a cellular system. However, we did not determine whether these lysine mutants had the same functions as wild-type pVHL.

The effect of missense mutations in *VH*L gene was examined by impairing E3 ligase activity. The E3 ubiquitin ligase complex, named as the VBC complex, is composed of elongin C, elongin B, Rbx1, cul2 and pVHL, and it functions as a substrate recognition molecule [16]. Missense mutant pVHLs formed the VBC complex and directly ubiquitinated HIF-1 α in vitro even it is existed at RCC which is nearby Elongin binding site (Fig. 4). In fact, L158Q had very weaken interacting affinity with Elongin C. These data are collected from over expression system which might be different from endogenous expression level. Thus, the examined missense mutations did not impair the E3 ligase activity of pVHL. Instability is crucial for impairing the functionality of missense mutant pVHLs. Three missense mutants (V155A, L158Q, and Q164R) sustained E3 ligase activity as indicated by their ability for the VBC complex and degrade HIF-2 α thereby decreased the expression VEGF, Glut-1 and Epo, which are target genes of HIF-2α [40] which promotes tumor cell growth, invasion and regulates glucose metabolism [41]. Protein levels of missense mutant pVHLs were inversely correlated with HIF-2α and its functionality to cell growth (Fig. 5). Collectively in case of three missense mutations of *VHL* gene (V155A, L158Q, and Q164R), they had function of tumor suppressor if they were protected from degradation. With regards to VHL disease, missense mutation of VHL gene induced pVHL instability, and the loss of function of pVHL caused an increase in the cellular level of HIFs, which promoted cell growth. Thus, we tested whether UCP depletion could rescue VHL disease particularly in RCC (Fig. 6). Depletion of UCP increased protein level of mutant pVHL and inhibited cell growth in vitro. Since depletion of UCP showed relative higher inhibition the growth of cell expressing V155A mutant pVHL than the others, we used cell expressing V155A mutant pVHL for ex vivo experiment. The UCP level was decreased and the pVHL level was increased in the tumor tissues and pVHL induced the expression of fibronectin and E-cadherin but HIF-2α was decreased in tumor nodules. Tumor microenvironments were composed with heterogeneous cells and molecules [42]. pVHL enhanced extracellular matrix protein, thus prolonged the tissue morphology and inhibited tumor metastasis HIFs independently [17]. V155A pVHL missense mutant is not existed in nature yet. Thus these data give biological prospect of new missense mutation of *VHL* gene.

Consequently, UCP ubiquitinated missense mutant pVHLs (V155A, L158Q and Q164R) via proteasomal degradation. Therefore depletion of UCP can be the therapeutic method for type 2 VHL disease such as RCC.

## Conclusion

UCP polyubiquitinates and degrades missense mutant VHL protein in vitro and in cellular system. Thereby delpletion of UCP restored protein level of V115A, L158Q and Q164R missense mutant pVHLs and lacking all of lysine residues of pVHL provided greater stability. Missense mutant pVHLs ubiquitinated ODD domain of HIF-1α in vitro. These mutant pVHL regulated target genes of HIF-2α thereby inhibited cell growth in 786-O cells depend on expression levels of them. Adenovirus-mediated shUCP delivery restored missense mutant pVHLs in vitro. It inhibited cell growth in vitro and inhibited tumor growth in a 786-V155A-expressing cell in ex vivo xenograft model. These data suggest that targeting of UCP can be one of therapeutic method in type 2 VHL disease caused by instability of pVHL missense mutants.

## Additional files

Additional file 1: Figure S1. Instability of missense mutant VHL proteins was caused by UCP. (A) Schematic diagram of missense mutation of VHL used in this study. (B) Seven recombinant protein of pVHL missense mutants were ubuiquitinated by UCP in vitro. Ubiquitinated forms were detected by western blot with Flag antibody. GST tagged recombinant proteins of mutant pVHLs were ubiquitinated by UCP in vitro. Indicated GST-pVHL and/or His-UCP protein were incubated at 37°C in the presence of E1 and Flag-ubiquitin. GST-pVHL polyubiquitination was detected by western blot with Flag antibody. (TIFF 322 kb) Additional file 2: Figure S2. Missense mutant pVHL constitute E3 ubiquitin complex in cell. (A) HEK293T cells were transfected with plasmid expressing GST tagged hotspot mutant pVHL and/or Flag tagged HIF-1α protein and then detected the interaction using GST-pull down assay. (B) HEK293T cells were transfected with plasmid expressing HA tagged mutant pVHL and/or GST tagged Elongin C protein and then detected the interaction using GST-pull down assay. Interacted mutants VHL proteins were detected by immunoblot as indicated. (TIFF 201 kb) Additional file 3: Figure S3. Ubiquitination is decreased in lysine zero mutant pVHL in cells significantly. (C) Single-lysine (K159, K171, and K196) and lysine-zero mutant pVHLs were analyzed in vitro ubiquitination assay. GST-tagged VHL was incubated with E1, Flag-ubiquitin and His-UCP at 37 °C for 1 h. Each GST-VHL was pulled-down with glutathione sepharose beads and analyzed by anti-GST or anti-HA antibody. (TIFF 115 kb) Additional file 4: Figure S4. RCC related missense mutant pVHL regulate HIF-2α target gene in mRNA level. (A) Conventional RT-PCR for HIF-2α target genes. Missense mutant *VHL* expressing 786-O stable cell line at 48 h after seeding and it was used for quantitation of transcripts of Glut-1, VEGF and Epo. (B) Calculation of intensity of band of Fig (A). (TIFF 190 kb) Additional file 5: Table S1. Primer sequences for RT-PCR and qRT-PCR. (TIFF 67 kb)

## Abbreviations

UCP: E2-EPF Ubiquitin carrier protein
pVHL: Von Hippel-Lindau protein
Ub: Ubiquitin
HIF: Hypoxia-inducible factors
RCC: Renal cell carcinoma
PBS: Phosphate buffered saline
GST: Glutathione S transferase
ODD: Oxygen dependent degradation
ATP: Adenosine triphosphate
DMEM: Dulbecco’s modified Eagle’s medium
FBS: Fetal bovine serum
DTT: Dithiothreitol
BL21: *Escherichia coli* BL21
EDTA: Ethylenediaminetetraacetic acid
IPTG: Isopropyl β-D-1-thiogalactopyranoside
NP-40: Nonyl phenoxypolyethoxylethanol
PMSF: Phenylmethanesulfonylfluoride
SDS-PAGE: Sodium dodecyl sulphate-polyacrylamide gel electrophoresis
PVDF: Polyvinylidene fluoride
VEGF: Vascular endothelial growth factor
Glut1: Glucose transporter 1
