# Supplementary figures and images for: E2-EPF UCP regulates stability and functions of missense mutant pVHL via ubiquitin mediated proteolysis
Source: BMC Cancer. 2015 Oct 26;15:800. doi: 10.1186/s12885-015-1786-8 (PMC4624580; doi:10.1186/s12885-015-1786-8)

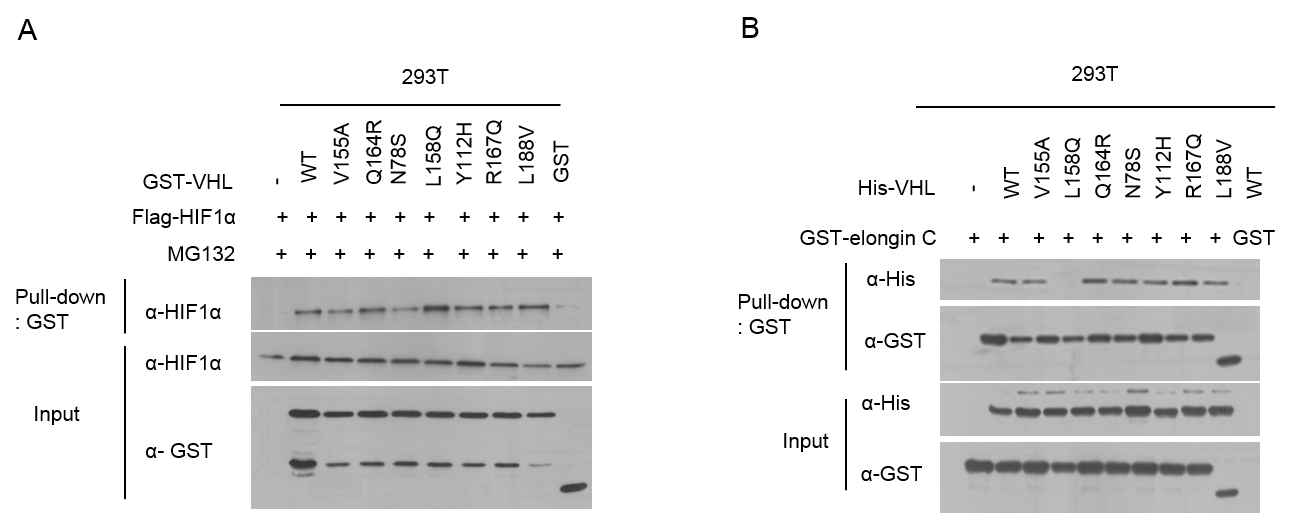

Supplement: Additional file 2: Figure S2. — Missense mutant pVHL constitute E3 ubiquitin complex in cell. (A) HEK293T cells were transfected with plasmid expressing GST tagged hotspot mutant pVHL and/or Flag tagged HIF-1α protein and then detected the interaction using GST-pull down assay. (B) HEK293T cells were transfected with plasmid expressing HA tagged mutant pVHL and/or GST tagged Elongin C protein and then detected the interaction using GST-pull down assay. Interacted mutants VHL proteins were detected by immunoblot as indicated. (TIFF 201 kb) [file 12885_2015_1786_MOESM2_ESM.tif]

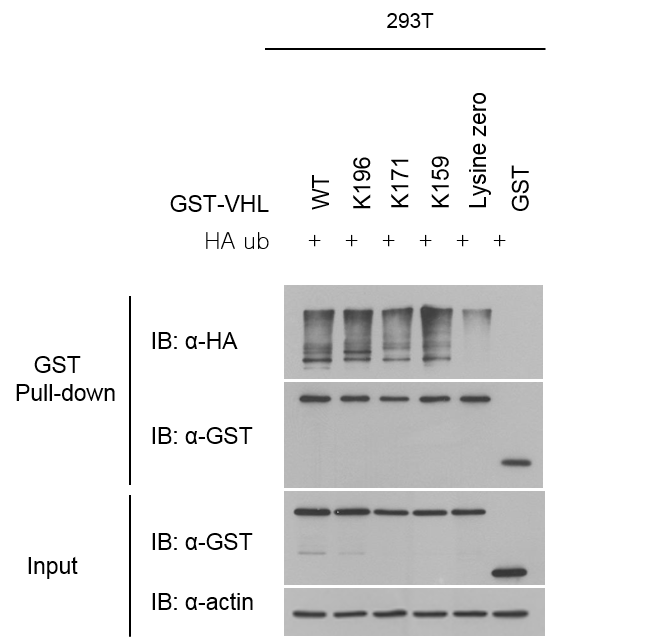

Supplement: Additional file 3: Figure S3. — Ubiquitination is decreased in lysine zero mutant pVHL in cells significantly. (C) Single-lysine (K159, K171, and K196) and lysine-zero mutant pVHLs were analyzed in vitro ubiquitination assay. GST-tagged VHL was incubated with E1, Flag-ubiquitin and His-UCP at 37 °C for 1 h. Each GST-VHL was pulled-down with glutathione sepharose beads and analyzed by anti-GST or anti-HA antibody. (TIFF 115 kb) [file 12885_2015_1786_MOESM3_ESM.tif]

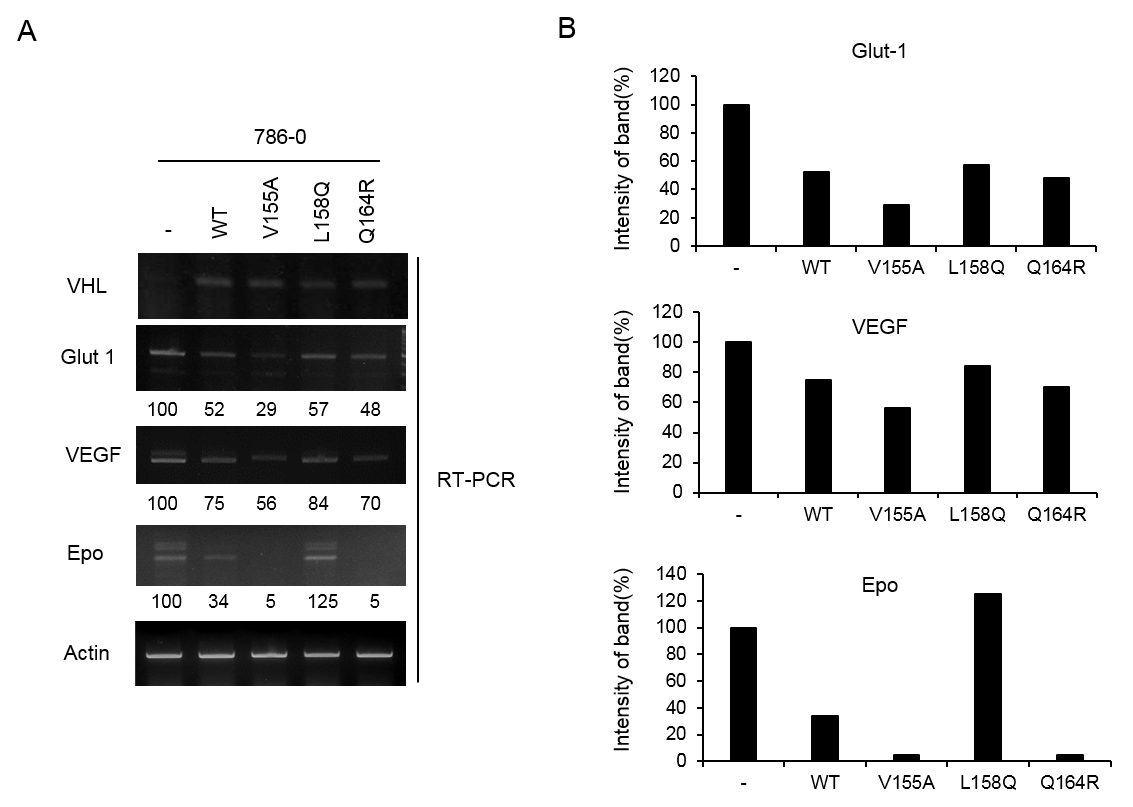

Supplement: Additional file 4: Figure S4. — RCC related missense mutant pVHL regulate HIF-2α target gene in mRNA level. (A) Conventional RT-PCR for HIF-2α target genes. Missense mutant VHL expressing 786-O stable cell line at 48 h after seeding and it was used for quantitation of transcripts of Glut-1, VEGF and Epo. (B) Calculation of intensity of band of Fig (A). (TIFF 190 kb) [file 12885_2015_1786_MOESM4_ESM.tif]

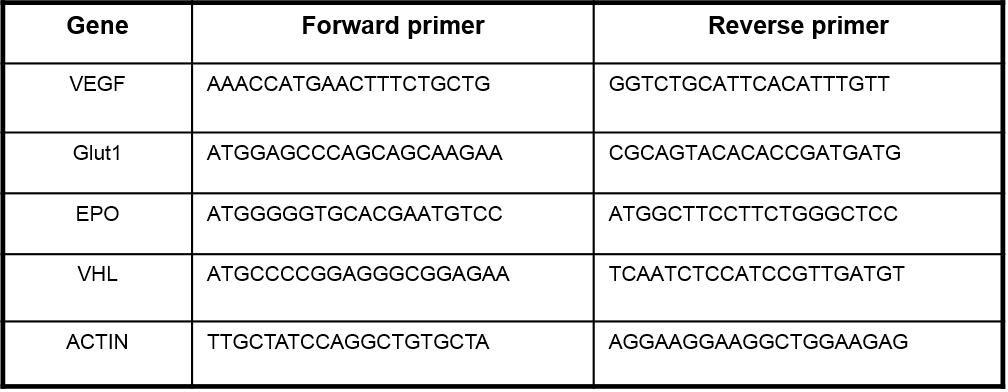

Supplement: Additional file 5: Table S1. — Primer sequences for RT-PCR and qRT-PCR. (TIFF 67 kb) [file 12885_2015_1786_MOESM5_ESM.tif]
